# Urban sewage sludge stabilization by alkalization-composting-vermicomposting process: Crop-livestock residue use

**DOI:** 10.1371/journal.pone.0289362

**Published:** 2023-09-07

**Authors:** Luiz Carlos Floriano da Silva, Luís Carlos Vinhas Ítavo, Ricardo Martins Santos, Camila Celeste Brandão Ferreira Ítavo, Vanessa Zirondi Longhini, Alexandre Menezes Dias, Gelson dos Santos Difante, Angelo Herbet Moreira Arcanjo, Juliana Caroline Santos Santana, Antonio Leandro Chaves Gurgel, Flavia de Oliveira Scarpino van Cleef

**Affiliations:** 1 Catholic University Dom Bosco (UCDB), Campo Grande, Brazil; 2 College of Veterinary Medicine and Animal Science (FAMEZ), Federal University of Mato Grosso do Sul (UFMS), Campo Grande, Brazil; 3 *Campus* Professora Cinobelina Elvas (CPCE), Universidade Federal do Piauí (UFPI), Bom Jesus, Brasil; 4 Center for Nuclear Energy in Agriculture, University of São Paulo, Piracicaba, Brazil; Gifu University, JAPAN

## Abstract

Waste management practices are vital for human health and the environment in a world where natural resources stress is expected to increase with the growth of population. Our study aimed to evaluate the potential use of crop-livestock residue as a bulking agent associated with the ideal level of hydrated lime for the stabilization and sanitization of urban sewage sludge through the alkalization-composting process. Therefore, we determined the alkalization efficiency on the heavy metal concentration in urban sewage sludge, quantified the viable eggs of helminths in pure and alkalized sludge, and measured the rate of earthworms (*Eisenia fetida*) surviving in the vermicomposting process using different levels of alkalized urban sewage sludge associated with crop-livestock residue. Four sequential trials were carried out in a completely randomized design with three replicates. The lime alkalization reduced the levels of Ba, As, Pb, Cu, Cr, Mo, Ni, and Zn compared to the pure urban sewage sludge. Using 30% w/w of lime in the urban sewage sludge (SS-30) for composting process reduced the viable helminth eggs by 71, 72, and 69% for sugarcane bagasse (*Saccharum officinarum*; SB), fresh chopped Napier-grass (*Pennisetum purpureum*; NG), and bovine ruminal content (BR), respectively. The ideal level of hydrated lime for stabilization and sanitization of urban sewage sludge was found to be 30%, which was able to reduce the heavy metals. The residues have the potential as a bulking agent for the composting of urban sewage sludge when associated with alkalization. The lime alkalization decreases the total number of helminth eggs and the number of viable eggs. The possibility of starting a vermicomposting using the mixtures is promising, evidenced by the earthworm survival in composting urban sewage sludge mixed with crop-livestock residues after 45 days of composting. The earthworm survival is maintained by an association of at least 80% of the crop-livestock residues.

## Introduction

Industry and urban wasting are of great concern with the growth of population, predicted to reach 9 billion people by 2050 [1]. Besides, the improvement in purchasing power has led to an increase in the consumption of goods posing greater challenges on the management of waste and its impact on the environment. In this context, the use of practices that minimize the anthropic effects on sustainability of ecosystems is a worldwide goal. Amongst them, vermicomposting is a tool to waste management treatment to reduce heavy metal content in the environment, which is a plan of the Sustainable Development Goals (SDG) that must be met by 2030 [1].

Urban sewage sludge may contain heavy metals and organic and inorganic substances that affect the environment and human health [2]. However, there is a potential for agricultural use of urban sewage sludge after treatment [3,4], such as elevated temperatures due to biological processes from composting and vermicomposting [5], thermal drying, solarization, gamma radiation, and the addition of lime or other alkaline substances [6]. In Brazil, the biosolids production and use of urban sewage sludge must meet the criteria and parameters established by CONAMA (Brazilian Environment Commission—Conselho Nacional de Meio Ambiente) in resolution 498/2020 [7].

Méndez et al. [8] determined that alkaline stabilization may be an alternative for controlling pathogens in the urban sewage sludge. This is because the addition of an alkaline agent, such as lime, for at least two hours, increases the pH up to 12 and maintains it constant for several days [9–11], altering the colloidal nature of pathogenic microorganisms’ cellular protoplasm lethally [2]. In an agricultural context, alkalization with hydrated lime solubilizes structural carbohydrates and lignin from sugarcane (*Saccharum officinarum*) [12], caused by the thermo-decomposition process, verified by the energy release [13]. These processes speed up the mineralization of organic compounds during vermicomposting, while rapid changes in pH do not allow bacteria to adapt to this environment, impacting growth and spore formation and promoting its lethality [14].

The accumulation of heavy metals in the soil due to the successive applications of urban sewage sludge is one of the concerns regarding the environmental safety and feasibility of using this residue in agriculture. The most frequent elements found in urban sewage sludge are barium (Ba), sodium (Na), potassium (K), arsenic (As), selenium (Se), mercury (Hg), antimony (Sb), silver (Ag), vanadium (V), cobalt (Co), aluminum (Al), boron (B), cadmium (Cd), calcium (Ca), lead (Pb), copper (Cu), chrome (Cr), sulfur (S), iron (Fe), phosphorus (P), magnesium (Mg), manganese (Mn), molybdenum (Mo), nickel (Ni), and zinc (Zn). Some of these nutrients, such as Mn, Fe, Mn, Ni, and Zn, are essential for nitrogen (N)-fixing bacteria and plants, because they participate in the structural and metabolic function acting in the protein stabilization and activation of enzymes [15].

According to Menon et al. [16], heavy metals depict variations in their bioaccumulative capacity that can be attributed to differences in their background levels, site differences, and the rate of absorption and elimination potential of a species. The authors recommended an environmental management program to reduce the toxicity risk to the human population. Joris et al. [17] determined that the alkalization altered the dynamics of Cu, Zn, Cd, and Ni, due to adsorption. Likewise, Huang et al. [18] studying the heavy metal resistance of earthworms, observed significant decreases in biomass and juvenile hatching. Soil metal solubility and bio-accessibility were essential factors that may have influenced the toxicity, thereby, earthworm generations under persistent exposure to soil metals could develop metal resistance. The earthworms were capable to transform solid wastes into enriched manure acting as viable biological agents [19], as they neutralize toxic metals with a small cysteine-rich metal-binding protein, metallothionein [20].

There is a crescent demand for sustainable technologies for reducing toxic heavy metals from contaminated biomass and soils [20]. The final product of the vermicomposting process for agricultural purposes results in environmental benefits, such as improved physical and chemical soil characteristics and water quality. Organic compost is a stable organic substrate that has undergone organic bio-oxidized to produce an organic and exothermic substance. The most significant factors influencing organic material degradation as a biological process are aeration, temperature, and humidity [5]; In addition, the concentrations of carbon, nitrogen, and phosphorus, as well as their relationships in the soil, also influence the processes of degradation of organic matter.

The use of earthworm species to neutralize heavy metals have already been studied by several authors [19,20]. Kızılkaya and Türkay [21] evaluated vermicomposting with *Eisenia fetida* across 90 d to stabilize sewage sludge mixed with different proportions of hazelnut husk and cow manure. Authors found a concentration of 15,962 mg/kg for Zn, 392 mg/kg for Cu, 10 mg/kg for Cd, 120 mg/kg for Pb, 111 mg/kg for Ni, and 718 mg/kg for Cr in the sewage sludge pure, when mixed with different proportions of bulking agent, it was verified a range of reduction of up to 98.3% for Zn, 32.0% for Cu, 81.8% for Cd, 84.3% for Pb, and 79.2% for Ni. Authors recommended mixtures in the proportion of 20 to 30% of sewage sludge with 35 to 40% of hazelnut husk and 35 to 40% of cow manure. Additionally, Ganguly and Chakraborty [22] demonstrated a reduction of heavy metals content using vermicomposting of sludge mixed with cow feces and straw (ratio 5:4:1), by adding *Perionyx excavatus*. The survival of earthworms was used as an indicator of the soil biological health quality [23], while the use of organic substrates, such as sugarcane bagasse and animal excreta, would favor the earthworm growth [24,25].

If on one hand, organic substrates are used as a conditioner for vermicomposting to mitigate the adverse effects of other raw materials (urban sewage sludge) on the survival of the earthworms. On the other hand, other wastes from the agricultural industry, such as bovine rumen content, is a residue discarded in slaughterhouses usually without any previous treatment with a potential to be used in vermicomposting. Additionally, using slaughterhouse wastes to produce biogas is an option to generate new jobs and reduce the transmission of zoonotic diseases [26]. However, studies using rumen content in urban sewage sludge treatment are scarce.

We hypothesized that agricultural wastes have the potential to be used in urban sewage sludge as a bulking agent for the composting process; moreover, the hydrated lime could be used to stabilize and sanitize the urban sewage sludge through the alkalization process. Therefore, our study aimed to use a crop-livestock residue [sugarcane bagasse (SB), fresh chopped Napier-grass (*Pennisetum purpureum*; NG), and bovine ruminal content (BR)],as a bulking agent associated with the ideal level of hydrated lime. Thus, we evaluated the alkalization efficiency on the heavy metal concentration in urban sewage sludge, quantified the viable eggs of helminths in pure and alkalized sludge, and determined the survival rate of earthworm (*Eisenia fetida*), using different levels of urban alkalized sewage sludge associated with the crop-livestock residue.

## Material and methods

### Ethical approval

Procedures in the current study involved only invertebrate organisms (Phylum Annelida), which according to Law No. 11,794 of October 08, 2008 [27], does not require an approval from Institutional Animal Care and Use Committee. The bovine ruminal content was obtained from a local slaughterhouse.

### Experimental site and sludge sampling

**Fig 1:**
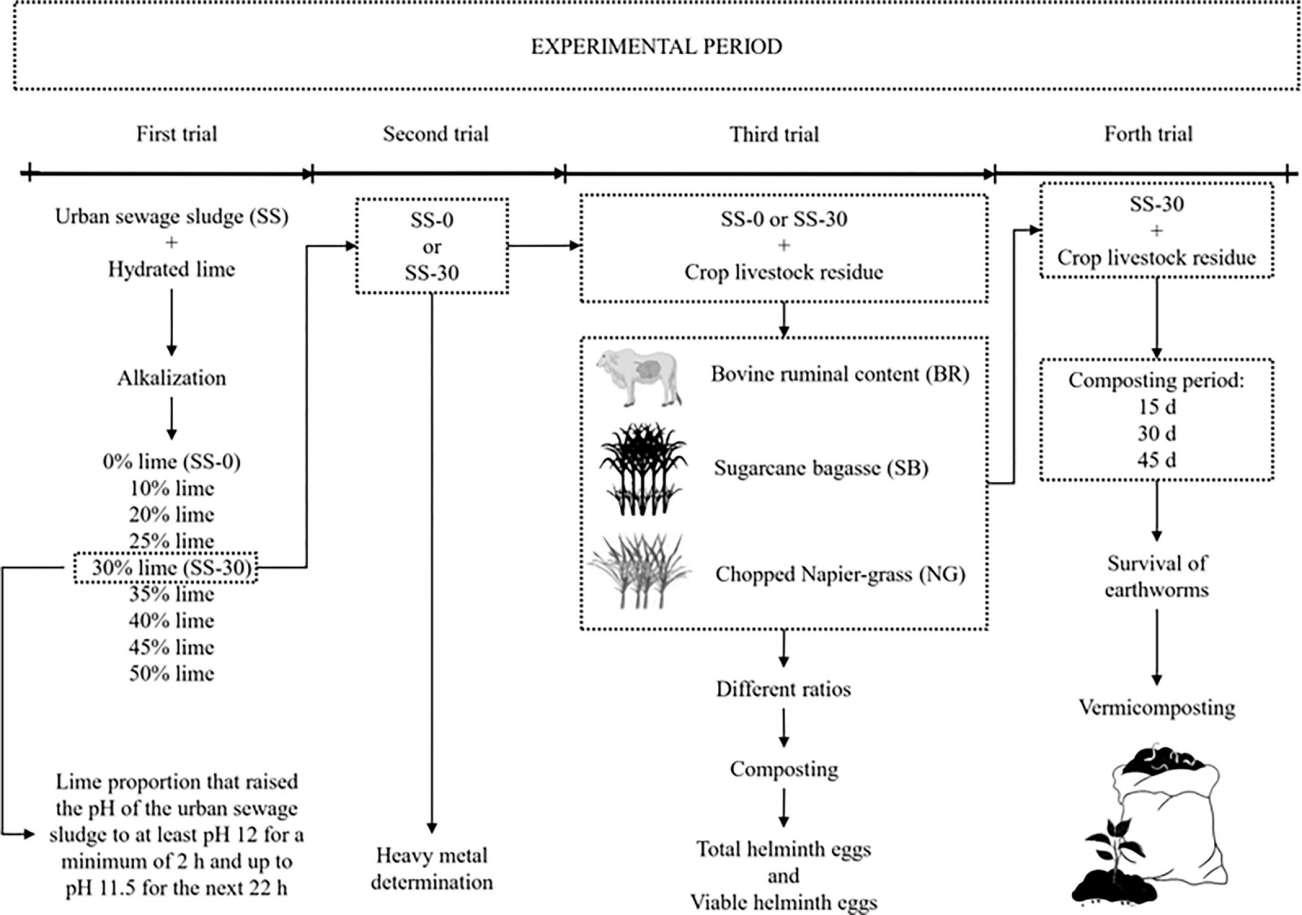
Schematic representation of the sequential experimental period.

The experiment was conducted in four sequential trials (Fig 1). In the first trial, lime levels were tested to determine the best level at which alkalization and pH stabilization of the urban sewage sludge (SS) occurred. In the second trial, the best level was compared with the compost without adding lime and the levels of heavy metals were determined. In the third trial, different substrates [sugarcane bagasse (SB), fresh chopped Napier-grass (*Pennisetum purpureum*; NG), and bovine ruminal content (BR)] were added to the compost with or without lime to verify the survival and number of helminth eggs. In the fourth trial, the alkaline treatment added to the substrates was used to verify the viability of these alkalized substrates on the survival of earthworms for vermicomposting.

Batches of urban sewage sludge (SS) were collected from wastewater treatment plants in Cardoso, Sao Paulo, Brazil. Four trials (Fig 1) were conducted at the State Center of Technological Education Paula Souza (CEETEPS), Technical State School Dr. José Luiz Viana Coutinho, located in Jales, SP, Brazil (20°16’6"S; 50°32’56"W, 486 m asl). The SS was dried through solarization inside agricultural greenhouses covered by clear plastic. Subsamples of SS were sent to the Laboratory of Fertilizers and Residues from the Agronomic Institute of Campinas for analyses of chemical composition (Table 1). Total nitrogen was determined by the Kjeldahl method. The moisture and volatile solids were determined by the loss of mass at 500°C for 60 min.

**Table 1:** Chemical composition of the pure urban sewage sludge (SS)^1^.

Parameter	SS
pH (in water 1:10)	7.2
Moisture at 60°C (% w/w)	4.6
Total dry solids (% w/w)	92.2
Volatile Solids (% w/w)	52.7
Organic Carbon (g/kg)	330
Nitrogen–Kjeldahl (g/kg)	45.5
C/N ratio	7.33
Ammonia Nitrogen (g/kg)	2.1
Nitrogen Nitrate-Nitrite (g/kg)	0.2
Potassium (g/kg)	0.8
Calcium (g/kg)	22.4
Sulfur (g/kg)	26.5
Phosphor (g/kg)	9.9
Magnesium (g/kg)	2.6

^1^ Anaerobic urban sewage sludge from Cardoso, Sao Paulo State, Brazil.

### First trial: Stabilization of urban sewage sludge by alkalization process

**Fig 2:**
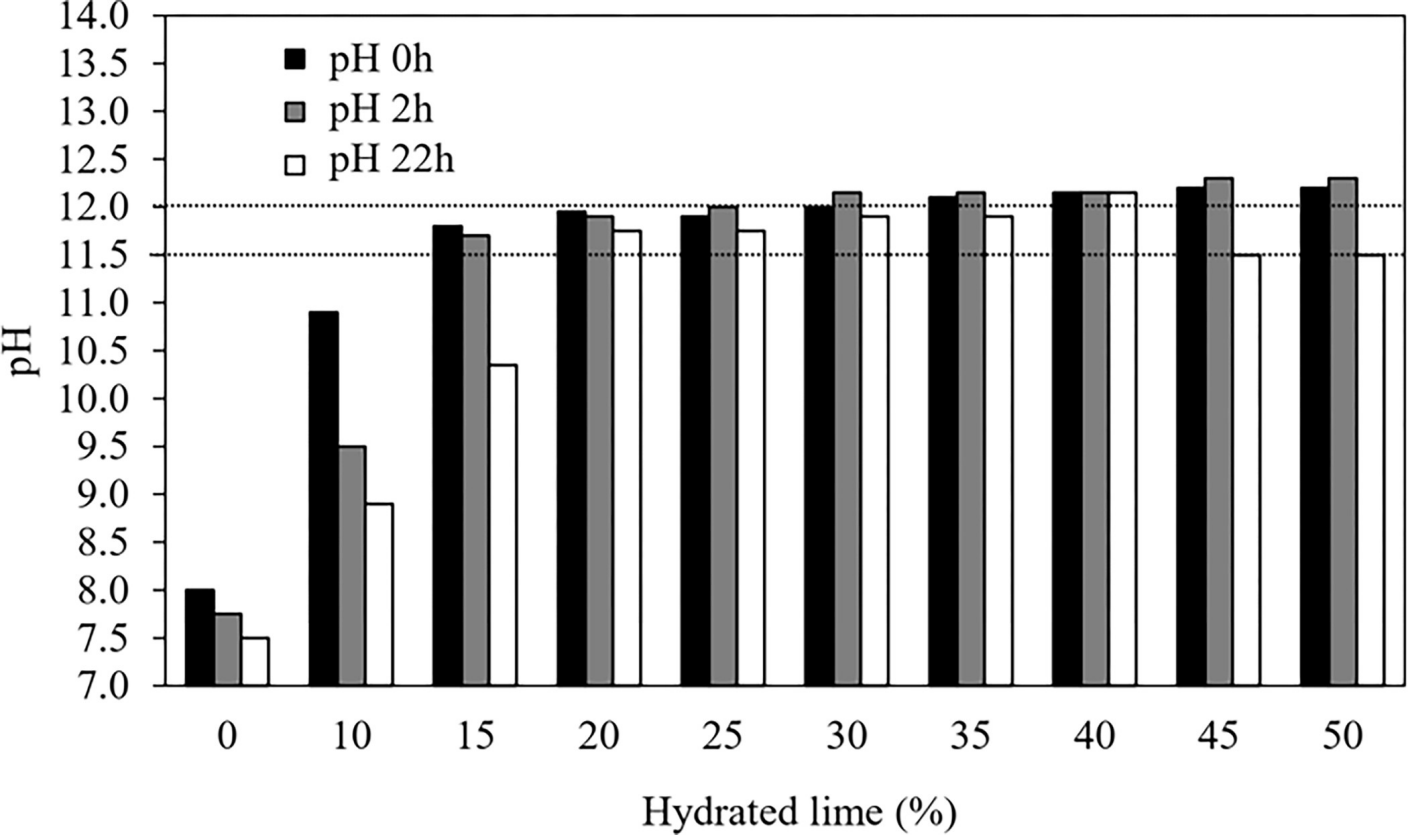
The pattern of pH of urban sewage sludge with increasing proportions of the hydrated lime. Dashed line: pH level used to choose hydrated lime proportion for urban sewage sludge alkalinization (for at least pH 12.0 after two h and up to pH 11.5 after 22 h).

Treatments were distributed in a completely randomized design and consisted of SS with ten increasing proportions of the hydrated lime (0, 10, 15, 20, 25, 30, 35, 40, 45, and 50% w/w). The dry weight of the urban sewage sludge was determined to calculate the lime mass to be added to 200 g of the mixture (Table 2). Measurements of pH were carried out on samples following recommendations of Andrade and Abreu [28]. The hydrated lime proportions that raised the pH of the urban sewage sludge to at least pH 12 for a minimum of 2 h and up to pH 11.5 for the next 22 h were chosen for subsequent trials (Fig 2).

**Table 2:** Proportions of the hydrated lime included in urban sewage sludge (SS).

Lime proportion	Lime mass	SS	SS Fresh^2^
(%)	(g)	(g DW^1^)	(g)
0	0	200	267.06
10	20	180	240.35
15	30	170	226.99
20	40	160	213.65
25	50	150	200.30
30	60	140	186.95
35	70	130	173.59
40	80	120	160.24
45	90	110	146.88
50	100	100	133.53

^1^ DW: Dry weight.

^2^ 25.11% of moisture.

### Second trial: Stabilization of urban sewage sludge by alkalization process

Treatments were distributed in a completely randomized design and consisted of pure urban sewage sludge (SS-0) or with 30% w/w of the hydrated lime (SS-30). Samples were analyzed for heavy metal concentration (Ba, As, Se, Hg, Cd, Pb, Cu, Cr, Mo, Ni, and Zn) following the methods SW 846 3051A [29] and SW 846 6010C [30]. The results were compared to the maximum limits of contaminants allowed in the urban sewage sludge [7] and are described in Table 3.

**Table 3:** Heavy metal concentration (mg/kg) in pure urban sewage sludge and urban sewage with alkaline treatment in comparison with legislation limits.

Parameters^1^	SS-0	SS-30	Variation	Limit Value^2^
Barium (Ba)	209.0 a	162.0 b	-22.5	1300.0
Arsenic (As)	2.5 a	< 1.0 b	-60.0	41.0
Selenium (Se)	2.1 a	1.6 b	-23.8	36.0
Mercury (Hg)	< 1.0 a	< 1.0 a	-	17.0
Cadmium (Cd)	1.4 a	1.7 a	+21.4	39.0
Lead (Pb)	30.0 a	24.6 b	-18.0	300.0
Copper (Cu)	214.0 a	168.0 b	-21.5	1500.0
Chrome (Cr)	1000.0 a	64.6 b	-93.5	1000.0
Molybdenum (Mo)	50.0 a	1.6 b	-96.8	50.0
Nickel (Ni)	420.0 a	12.1 b	-97.1	420.0
Zinc (Zn)	2800 a	990.0 b	-64.6	2800.0

^1^ Means followed by different lowercase letters in the same row differ by Tukey test (P<0.05).

^2^ Brazil [7]. SS-0: Pure urban sewage sludge (control: Without lime); SS-30: Urban sewage sludge plus 30% lime.

### Third trial: Stabilization of urban sewage sludge by composting process

Treatments were distributed in a completely randomized design with a factorial arrangement: urban sewage sludge [pure (SS-0) or with 30% w/w lime (SS-30)] and three crop-livestock residues (SB, NG and BR), as bulking agents for the composting process. The crop-livestock residue was added in increasing ratios to the urban sewage sludge (20, 40, 60, and 80% w/w). The composting process was conducted according to Sundberg [31]. Eighteen liter-plastic buckets were filled with the mixtures for the composting and arranged on shelves protected from sun and rain. The moisture of the mixtures for composting was corrected to 60%, which was maintained through manual irrigation. Samples were collected 21 d after the mixture for composting. A composite sample of 1000 g was homogenized, placed in sterile plastic bags, and sent for microbiological and parasitic analyses.

### Fourth trial: Stabilization of urban sewage sludge by vermicomposting process

Vermicomposting was carried out in plastic containers of 1 L capacity with a perforated cap to avoid sunlight and the escape of the earthworms. The substrate consisted of urban sewage sludge hydrated with 30% w/w of lime (SS-30) mixed with three crop-livestock residues (SB, NG, and BR). The crop-livestock residues were each added as bulking agents in ten increasing ratios (10, 20, 30, 40, 50, 60, 70, 80, 90, and 100% w/w). After 15, 30, and 45 d of the composting process, 700 ml of each substrate was filled to the container. At reproductive age, five adults of *Eisenia fetida* were added to each composting substrate, since it can neutralize heavy metals [20]. An acute toxicity bioassay was adapted following ISO 11268–1 [32] and ISO 11628–2 [33] to identify the survival of earthworms to the treatments for the vermicomposting process.

### Statistical analysis

Treatments in all four trials were distributed in a completely randomized design with three replicates. Data were analyzed as a completely randomized design using the GLM procedure of the SAS statistical package (SAS University Edition, SAS Institute Inc. Cary, CA, USA). One-way ANOVA was used to determine the effects of treatment on the heavy metal concentration, total helminth eggs, viable helminth eggs, and survival of earthworms from the analysis test, and means were compared using Tukey test at 5% significance. The total helminth and viable helminth eggs variables were analyzed by orthogonal polynomial regression using the SAS GLM procedure in function of the different ratios of urban sewage sludge (SS) and crop-livestock residues. The models were chosen based on the significance of the regression coefficients using the F test and the coefficient of determination.

## Results

### Stabilization of urban sewage sludge by alkalization process

The pure urban sewage sludge showed a concentration of ammonia-N 2.1 g/kg, and 0.2 g/kg Nitrogen Nitrate-Nitrite. The macro-minerals observed were 0.8 g/kg of K, 22.4 g/kg of Ca, 26.5 g/kg of S, 9.9 g/kg of P, and 2.6 g/kg of Mg (Table 1). The hydrated lime proportions that could raise the pH of the urban sewage sludge for pH of at least 12 for a minimum of 2 h and up to pH 11.5 for the next 22 h was the urban sewage sludge with 30% of hydrated lime showed a pH above 12, recorded in two hours, and stabilized at 22 h (Fig 2).

There was a reduction of most of the heavy metal concentration (Ba, As, Se, Pb, Cu, Cr, Mo, Ni, and Zn) in the urban sewage sludge with the addition of 30% w/w of hydrated lime (P<0.05), but no effects on concentration of Hg and Cd (Table 3). The SS-30 did not exceed the maximum limits of contaminants allowed in the urban sewage sludge by the current Brazilian regulations [7].

### Stabilization of urban sewage sludge by composting process

There was a linear effect of increasing the proportion of SS-0 in the total number of helminth eggs and the viable helminth eggs in the mixture after 21 d of composting (P<0.05; Table 4) on SB, NG, and BR treatments. Likewise, there was a linear effect of SS-30 on BG and BR to total helminth eggs number. For the viable helminth eggs variable, there was no effect of increasing the proportion of only SS-0 in the mixture after 21 d of composting (P≥0.05) on SB. For other crop-livestock residues, there was a linear effect of both SS-0 and SS-30.

**Table 4:** Total helminth eggs and viable helminth eggs in different ratios of urban sewage sludges (SS) and crop-livestock residues as substrate after 21 d of the composting.

Total helminth eggs (N°/g TS) ^1^								
Ratio	20:80	40:60	60:40	80:20	R^2^	Equation		
SS:SB								
SS-0	70 a	75 a	102 a	96 a	0.704	Y = 59.50 + 0.5250×level of SS		
SS-30	24 b	40 b	39 b	59 b	0.856	Y = 14.50 + 0.5200×level of SS		
SS:NG								
SS-0	62 a	78 a	93 a	103 a	0.942	Y = 49.50 + 0.6900×level of SS		
SS-30	39 b	43 b	46 b	38 b	-	Y = 42		
SS:BR								
SS-0	89 a	102 a	109 a	116 a	0.823	Y = 82.00 + 0.4400×level of SS		
SS-30	27 b	50 b	51 b	56 b	0.749	Y = 24.00 + 0.4400×level of SS		
Viable helminth eggs (N°/g TS)								
SS:SB								
SS-0	58 a	58 a	83 a	56 a	-	Y = 64		
SS-30	8 b	13 b	19 b	31 b	0.946	Y = -1.00 + 0.3750×level of SS		
SS:NG								
SS-0	54 a	61 a	71 a	90 a	0.908	Y = 39.50 + 0.5900×level of SS		
SS-30	17 b	18 b	21 b	19 b	0.365	Y = 16.50 + 0.0450×level of SS		
SS:BR								
SS-0	71 a	80 a	71 a	97 a	0.482	Y = 62.50 + 0.3450×level of SS		
SS-30	11 b	24 b	34 b	29 b	0.688	Y = 8.50 + 0.3200×level of SS		

^1^ N°/g TS: Number of eggs per gram of total solids.

SS-0: Pure urban sewage sludge (control: Without lime); SS-30: Urban sewage sludge plus 30% lime; SB: Sugarcane bagasse; NG: Chopped Napier-grass; BR: Bovine ruminal content.

Means followed by different lowercase letters in the same column differ by Tukey test (P<0.05).

The SS alkalization with 30% w/w of hydrated lime decreased the total number of helminth eggs and the number of viable eggs after 21 d of the composting, regardless of the proportion of crop-livestock residues (P<0.05; Table 4). Using lime in the composting process reduced the total number of helminth eggs by 53, 49, and 56%, while the viable helminth eggs were decreased by 71, 72, and 69% for SB, NG, and BR, respectively. The number of viable eggs represented 74, 82, and 77% of the total number of helminth eggs in the mixture of pure urban sewage sludge with SB, NG, or BR, respectively. On the other hand, for SB, NG, or BR associated with lime alkalization, the number of viable eggs represented 44, 45, and 53% of the total number of helminth eggs, respectively.

### Stabilization of urban sewage sludge by vermicomposting process

The earthworm survival rate was zero on day 15 of the composting process, regardless of the crop-livestock residue or level of inclusion in the SS-30. When the earthworms were added to the mixtures, their behavior was observed to be highly agitated, with frantic movement toward the material’s periphery and the pot wall surface. In less than 30 minutes, the earthworms died in the treatments with a high concentration of urban sewage sludge (>70%), and after 2 h, a 100% of mortality was found. In addition, there was no earthworm survival rate in the mixture with ratios above 30% of urban sewage sludge alkalized. For this reason, we did not include these data in Table 5. On day 30, there was an earthworm survival rate of 33.3% and 11.1% for the composting with 10% of urban sewage sludge alkalized plus 90% of SB or BR, respectively (P<0.05; Table 5). While the earthworm survival in the NG treatment only occurred with the inclusion of 0% of the urban sewage sludge alkalized. On the other hand, by increasing the composting time to 45 d, the earthworm survival rate increased for all crop-livestock residues, with 10% of the urban sewage sludge alkalized. In contrast, the treatment with a ratio of 20:80 of SS-30:BR increased to 100% the earthworm survival rate.

**Table 5:** Earthworm survival rate (%) in different ratios of urban sewage sludges alkalized with 30% of lime and crop-livestock residues over time of the composting process.

	SS-30:SB	SS-30:NG	SS-30:BR
30 d of the composting process^1^			
0:100	55.5 b	22.2 c	100.0 a
10:90	33.3 a	0.0 c	11.1 b
20:80^2^	0.0 a	0.0 a	0.0 a
45 d of the composting process			
0:100	100.0 a	100.0 a	100.0 a
10:90	66.6 b	22.2 c	100.0 a
20:80^2^	0.0 b	0.0 b	100.0 a

SS-30: Urban sewage sludge plus 30% lime; SB: Sugarcane bagasse; NG: Fresh chopped Napier-grass; BR: Bovine ruminal content.

Means followed by different lowercase letters in the same row differ by Tukey test (P<0.05).

^1^ There was no earthworm survival in the mixture on day 15 of the composting process.

^2^ There was no earthworm survival in the mixture with ratios above 30% of urban sewage sludge.

## Discussion

Reducing heavy metals from urban sewage sludge allows their use in agriculture without causing environmental damage. The CONAMA Resolution n° 498/2020 [7] establishes that the amount of alkaline material added to the urban sewage sludge must raise the pH to at least 12, for a minimum of 72 h. Along with the microbiological and parasitic aspects, the presence of heavy metals in urban sewage sludge is the primary concern of society regarding its use as an agricultural input. The levels of heavy metals determined in the analysis of both urban sewage sludge (SS-0 or SS-30) were below the maximum limits established in CONAMA Resolution n° 375/2006 [32]. This means that the agricultural use of sludge is safe for metals Cd, Pb, Cu, Cr, Ni, and Zn. However, in pure urban sewage sludge some of the heavy metals (Cr, Mo, Ni, and Zn) were at the maximum limits of contaminants allowed by the Brazilian legislation [7].

The lime raises the pH causing a reaction with heavy metals, forming a precipitate with the carbonate ion [34]. In addition, during the alkalization process, some of the heavy metals, such as Cu, Zn, Cd, and Ni may be reduced through adsorption [17]. This process would be recommended mainly in urban sewage sludge with high levels of toxic metals before preparing for vermicomposting.

Regarding pathogenic agents, the analysis of helminth eggs identified that the number of viable eggs was greater than the limits established by CONAMA [7] of<1.0 egg/g TS (number of eggs per gram of total solids). Our study showed that until day 21 of the composting, regardless of the crop-livestock residue, the urban sewage sludge could not be used in agriculture. Overall, the number of viable helminth eggs decreased from 71 to 20 eggs/g TS with the alkalization, regardless of the crop-livestock residue or level of inclusion. This finding demonstrates that alkalization promotes the sanitization of urban sewage sludge when the pH is increased.

The lime in the urban sewage sludge plays an important role in the inactivation pathogen by raising the pH (≥12) and temperature (in 75 min at >55°C) [35]. In our study, 30% of lime was enough to raise the pH to 12 for 2 h. Those rapid changes in the pH do not allow pathogenic microorganisms’ adaptation, affecting growth and promoting their lethality [2,14]. In addition, at higher pH means that more ammonium is being converted into ammonia, which is responsible to inactivation of the pathogenic organisms, such as *Ascaris* eggs [36,37].

Hydrated lime did not raise the temperature of the urban sewage sludge above 50°C (thermophilic temperatures). The average temperature in the urban sewage sludge mixed with crop-livestock residues was approximately 35°C. Mesophilic temperatures between 34 and 45°C, under aerobic conditions are sufficient to prevent the hatching of helminth eggs [38]. However, in this study, only on day 21 of composting temperatures greater than 40°C for the three crop-livestock residues were observed. According to Fernandes and Silva [39], low temperatures might occur in residue mixtures in the composting process because urban sewage sludge with a high degree of stabilization (50–60% of fixed solids) may not contain the energy nutrients indispensable to microbiology activity. Therefore, the disinfection process probably occurred by pH change and by the N transformation in ammonia in the urban sewage sludge.

The use of earthworms plays an important role in transforming urban sewage sludge and agricultural waste into biosolids. Wu et al. [40] reported that using urban plant litter as an additive in the vermicomposting of urban sewage sludge significantly reduced heavy metal content and increased the organic carbon content of the final compost. However, several studies have shown a negative impact of high concentrations of urban sewage sludge on earthworm development [24,41], but not with the level of lethality observed in the present study. According to Kızılkaya and Türkay [21], sewage sludge as raw material should be added between 20 to 30% for vermicomposting, because greater proportions would resulted in earthworm mortality above 60%, due to the high toxicity of heavy metals in earthworm tissues.

Alvarenga et al. [42] reported that the high concentrations of metals in the urban sewage sludge and the high electrical conductivity are responsible for the high toxicity to earthworms. In addition, at the beginning of the composting process, pH of the sludge was greater than 10, due to the lime application.

The fermentation of sludge facilitates the processes of genesis of acids and reduces methanogenesis. The greater the pH and the ammonia concentration, the greater free ammonia in the environment [43]. Ammonia associated with the high electrical conductivity and possibly the presence of some heavy metal in concentration lethal for earthworms drove the lethality observed until day 45. In this period, the mean pH was reduced to 8.2. It is considered that the habitat is limited when less than 20% of the organisms are found on the test substrate [44].

Bulking agent use in urban sewage sludge has shown positive responses in reducing toxicity and increasing the survival rate and earthworm density [45–47]. On the other hand, studies have suggested that pre-treatments must occur in the substrate before vermicomposting to eliminate toxicity agents [48]. In the present study, the composting time of fewer than 45 d does not guarantee high survival rates of the earthworms, even if urban sewage sludge alkalized is not added.

The 100% survival rate was verified with the bovine ruminal content, with a minimum participation of 80% in the compost. According to Ganguly et al. [47], high rates of organic carbon can reduce the earthworm population growth due to reduced acclimatization and greater cellular toxicity. Thus, crop-livestock residues such as sugarcane bagasse and fresh chopped Napier-grass may have contributed to the higher earthworm lethality than bovine ruminal content. Our study showed that mixtures of urban sewage sludge with 80% of BR and 90% of SB or NG do not fit as a substrate with “limited habitat function”.

## Conclusions

The ideal level of hydrated lime for stabilization and sanitization of urban sewage sludge was found to be 30%, in which was possible to reduce Ba, As, Se, Pb, Cu, Cr, Mo, Ni, and Zn in relation to the sludge pure sewage. The crop-livestock residues had potential as a bulking agents for the composting of urban sewage sludge when associated with alkalization, decreasing the total number of helminth eggs and the number of viable eggs after 21 days of composting, regardless of their proportion used as a substrate.

The possibility of starting a vermicomposting using the mixtures is promising, evidenced by the earthworm survival in composting urban sewage sludge mixed with crop-livestock residues after 45 days of composting. The earthworm survival is maintained by an association of at least 80% of the crop-livestock residues.
